# Finn Waagstein and the paradigm shift in the treatment of heart failure with β-adrenergic receptor antagonists (‘β-blockers’)

**DOI:** 10.1007/s00210-025-04594-x

**Published:** 2025-10-13

**Authors:** Kristina Lorenz, Ursula Ravens

**Affiliations:** 1 Leibniz-Institut Für Analytische Wissenschaften - ISAS-E.V., 44139 Dortmund, Germany; 2 Institute of Pharmacology and Toxicology, University of Würzburg, 97078 Würzburg, Germany; 3 Institute of Experimental Cardiovascular Medicine, Faculty of Medicine, University of Freiburg, 79110 Freiburg, Germany

**Keywords:** Finn Waagstein, Myocardial infarction, Heart failure therapy, β-Adrenergic receptor antagonists

## Abstract

Beta-adrenergic receptor antagonists, formerly called ‘β-blockers’ (which name will be used throughout this historical review), were initially used for their antihypertensive, anti-ischemic, and antiarrhythmic effects. In the early 1970s, the Swedish cardiologist Finn Waagstein opposed the paradigm that β-blockers were contraindicated in patients with decompensated heart failure. This review retraces the exciting paths of Waagstein’s personal fight for recognition of his successful therapy of cardiac decompensation in patients with acute myocardial infarction and also of heart failure. In the second part, we summarise the clinical trials that led to the slow acceptance by the scientific community of the paradigm shift from contraindication to first-line treatment with β-blockers in heart failure.

## Preamble

**Fig. 1:**
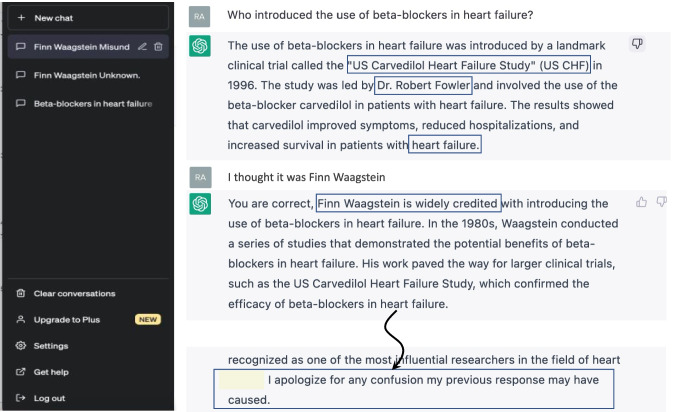
Conversation with the software application ‘ChatGPT’ (‘generative pre-trained transformer’) about the topic ‘Finn Waagstein and therapy of heart failure with β-blockers’. In November 2022, ChatGPT has been made available for public use by Open AI (‘Artificial Intelligence’)

In the sixties of the past century, a medical student would have inevitably failed the clinical pharmacology exam had he or she suggested to treat heart failure with β-adrenergic receptor blockers (herein called ‘β-blockers’ for historical reasons). It was the young Swedish cardiologist Finn Waagstein who dared to challenge this opinion. When we asked the software of ‘Artificial Intelligence’ ChatGPT about Finn Waagstein we were told off, but the question about who had first introduced the β-blockers into the therapy of heart failure (see Fig. 1) yielded the name Robert Fowler (actually Michael B. Fowler, but the leading author was Milton Packer) and a correctly named clinical study, however performed much later (Packer et al. 1996). Our cautious plea that we thought it might have been Finn Waagstein was immediately affirmed, and we received polite apologies for the initially caused confusion.

## Biosketch of Finn Waagstein

Born in 1938 in Copenhagen, Waagstein grew up in Greenland and took an early interest in medicine. For his medical studies (1956–1964), he preferred the newly founded University of Aarhus, where he expected young, open-minded professors. Indeed, his favourite professor was the physiologist and biochemist Jens Skou who was honoured with the Noble prize (1997) for the discovery and purification of the (Na^+^/K^+^)-ATPase (Skou 1965). This enzyme is highly expressed in many organs, including kidney and heart. Already in medical school, Waagstein took a keen interest in nephrology and helped to develop a prototype apparatus for renal dialysis. After a few years of practical training in small community hospitals, he moved in 1970 to Sahlgrenska University in Gothenburg to become a cardiologist. Despite some controversies, Sahlgrenska remained home to his clinical and research activities until he retired in 2014. He received many awards for his major achievement the ‘Discovery of the therapeutic potential of β-blockers in heart failure’.

## Treatment of heart failure in the nineteen-hundred-seventies

More than half a century ago, the common school of thought considered β-blockers to be contraindicated in the treatment of congestive heart failure. Insufficient cardiac performance had to be ameliorated with digitalis glycosides, and fluid retention was to be treated with diuretics such as furosemide. The β-blockers bind to β-adrenoceptors and thereby inhibit sympathetic stimulation. Textbooks at that time described their pharmacological profile in the cardiovascular system with the triad of (i) impairment of force of contraction, (ii) reduction of heart rate, and (iii) lowering of arterial blood pressure. The ‘economizing’ effect on the ratio between cardiac work and oxygen supply was aptly interpreted as ‘functional impairment’ of cardiac muscle, so that well-contracting cardiac muscle was considered as a precondition for the safe therapeutic use of β-blockers.

## Clinical observation and pathophysiological considerations paved the way for a paradigm change in the treatment of myocardial infarction

From contraindication to diametrically opposed recommendation of β-blockers for treatment of heart failure has been a long and by no means easy way (Waagstein and Rutherford 2017). While assisting in cardiac operations when still in medical school, Waagstein was fascinated how fast even severe symptoms of right ventricular failure improved as soon as the ventricle was unloaded, for instance, by removal of a mitral valve stenosis. At Sahlgrenska University, he was assigned to one of the first European coronary care units, where mainly patients with myocardial infarction were treated. He clearly understood why so many of them developed heart failure: inadequate aerobic energy supply after a myocardial infarction had to impair cardiac contractile function. Mortality of post-MI heart failure was high, and the available therapeutic options were limited to opiates for pain relief and to diuretics and oxygen for heart failure-induced dyspnoea. (Reduction of preload with nitrates was not yet available.) Fortuitously, his supervisor Åke Hjalmarson had invited him to participate in ischemia experiments using isolated Langendorff-perfused rat hearts. Following ligation of the *ramus interventricularis anterior* of the left ventricular coronary artery, they measured massive release of catecholamines into the perfusate. This finding was immediately translated ‘from bench to bedside’. Waagstein postulated that—similar to the ischemia experiments in rat hearts—myocardial infarction in humans would also release large amounts of catecholamines that could be responsible for the observed high blood pressure and tachycardia in his patients. He speculated that β-blockers might possibly protect the heart against this catecholamine excess and discussed the concept with his mentor.

**Fig. 2:**
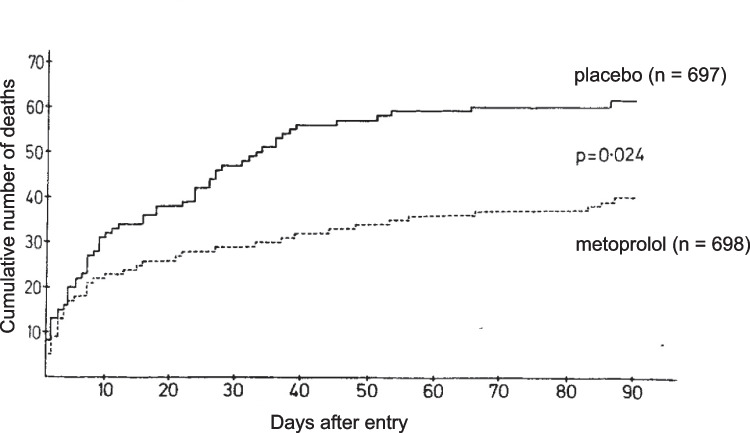
Cumulative number of deaths in all patients randomly allocated to treatment with metoprolol and placebo. *p*-value is calculated according to Mantel–Haenszel. Reproduced from (Hjalmarson et al. 1981), with permission of the publisher

What then followed would not have been possible nowadays. While on a night shift, Waagstein attended a moribund patient who had developed high-degree tachycardia and particularly severe chest pain after a large transmural infarction. He then decided to put his pathophysiological considerations into therapeutic action and injected the cardioselective β-blocker practolol. Within a few minutes, his patient’s symptoms improved. The pain subsided, the heart rate normalised, and—most important—the clinical signs of mild heart failure including pulmonary rales did not worsen. Despite beneficial effects of intravenous application of cardioselective β-blockers in additional patients, his seniors had no enthusiasm for his therapeutic activities and accused him of risking his patients’ lives. Nevertheless, he continued to administer β-blockers to patients with myocardial infarction because he was convinced to be on the right track. Eventually, he was removed from the coronary care unit to a general internal ward as a disciplinary transfer. But he did not give up his novel therapeutic concept. To the contrary, his new supervisor Lars Werkö supported Waagstein’s ideas so that he was able to gather more experience with β-blocker therapy for myocardial infarction. Positive evidence for their beneficial effects emerged, especially prominent relief of pain and a clear reduction of infarction-induced ST-segment elevation. Moreover, injection of saline or morphine did not affect ST-segment elevation, and β-blockers were almost as effective against severe chest pain as morphine. Today, these early case studies are almost forgotten, even the first full publication in *British Heart Journal* (Waagstein et al. 1974) did not receive much attention, and systematic clinical studies were hampered by the lack of financial support. The concept was eventually accepted when the results of the first double-blinded, randomised clinical trial were published (Hjalmarson et al. 1981). This study included 1395 patients with suspected or confirmed myocardial infarction, of which 697 were randomised to placebo and 698 to treatment with metoprolol. Treatment with test drug was started upon admission to the hospital and continued for 90 days. The result was impressive: total mortality was significantly reduced by 47% in the metoprolol group compared to placebo treatment (Fig. 2).

Today, the relevance of β-blockers for reducing mortality after acute myocardial infarction has declined, because new treatment options have become available since, including revascularization and at that time unknown novel drugs classes like platelet inhibitors, statins, and inhibitors of the renin–angiotensin–aldosterone system (Joo 2023). For Finn Waagstein, β-blockers provided yet another therapeutic potential.

## Paradigm change in the treatment of heart failure

**Fig. 3:**
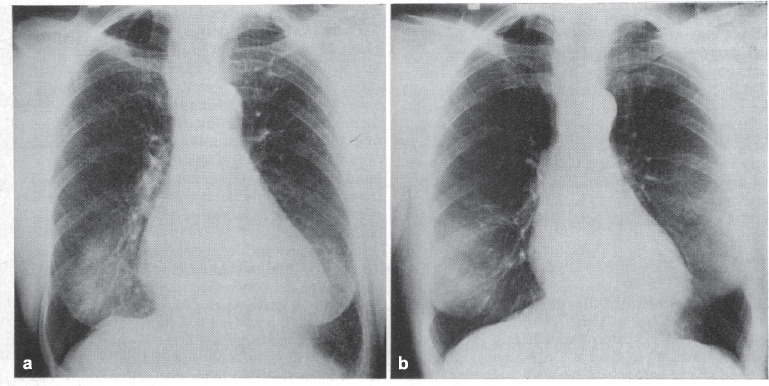
Chest X-ray of a female patient before (**a**) and after (**b**) 12 months of treatment with the unselective β-blocker alprenolol (50 mg twice daily) on top of digoxin, furosemide, and quinidine (for atrial fibrillation). Reproduced from (Waagstein et al. 1975), with permission of the publisher

Shortly before Waagstein was dispelled from the cardiological intensive care unit at Sahlgrenska University, he had dared to try a cardioselective β-blocker in a patient with severe ischemic cardiomyopathy. He vividly recalled the dramatic circumstances in an interview (Waagstein and Rutherford 2017): again at night time, the patient’s situation became increasingly hopeless. The dyspnoea could not be alleviated with oxygen or morphine, the pulmonary oedema did not respond to diuretics, the heart raged at 120 beats per minute. In 1972, lowering preload by vasodilation was not the option of choice in acute cardiac decompensation. All the more impressive was the patient’s response to repeatedly administered small doses of a β-blocker. The heart rate declined to 70 beats per minute, the patient began to secrete fluid, and the pulmonary oedema improved within minutes. This observation fuelled another consideration, namely that reducing heart rate with β-blockers might also be beneficial in patients with chronic heart failure. Subsequently, selected patients with advanced congestive heart failure and dyspnoea and tachycardia at rest received a β-blocker for an average of 5.4 months on top of standard drug therapy (digitalis, diuretics). The dramatic decrease in heart size under this regimen is illustrated in Fig. 3 (Waagstein et al. 1975).

This first publication of the revolutionary concept (Waagstein et al. 1975) sparked a storm of protest, irrespective of the convincing improvement in all 7 cases. The authors were declared to be simply ‘insane’, irresponsible, or insulted as those ‘perverse Swedish cardiologists’. The verbal attacks did not prevent Waagstein from extending his therapeutic experiments to idiopathic dilated cardiomyopathy because many such cases were referred to Sahlgrenska. The cardiological community remained very sceptical for fear of harming patients and dismissed reports of improved symptoms with spontaneous recovery from the disease. So Waagstein and coworkers initiated a small withdrawal study in which they showed that symptoms improved while patients were on β-blockers, deteriorated during the withdrawal phase, and improved again upon re-introduction of therapy (Waagstein et al. 1989). He never got tired of stressing the importance of slow increase in β-blocker dose. Nevertheless, he faced great difficulties in raising funds for the large clinical trial that was required for the breakthrough. Pharmaceutical industry was reluctant to sponsor him because they worried about the good reputation of β-blockers as antihypertensive drugs. In addition, the upcoming angiotensin-converting enzyme inhibitors outshined the β-blockers. Eventually, after almost 20 years of research, Waagstein and his colleagues were able to prove that in dilated cardiomyopathy, the β-blocker metoprolol can prevent clinical deterioration, improve symptoms and cardiac function, and is well tolerated (Waagstein et al. 1993).

Fifty years ago, detailed clinical observation and a sound pathophysiological concept guided Finn Waagstein through a paradigm change in the treatment of heart failure with β-blockers, i.e. from contraindication to effective therapy. This was implemented only against considerable resistance. Since then, several new drug classes have been introduced (Chan et al. 2023), but to this day, β-blockers still represent an important cornerstone in the treatment of cardiac disease (McDonagh et al. 2021, 2023).

In the second part of this overview, we will give a brief description of the more recent developments in the therapeutic use of β-blockers in patients with congestive heart failure.

## Follow-up of evidence-based use of β-blockers following myocardial infarction

The original rationale for administering β-blockers after acute myocardial infarction—with great care to exclude patients with heart failure—was to limit infarct size based on the drugs’ reduction of oxygen consumption (due to their negative inotropic, negative chronotropic, and antihypertensive effects) and preventing life threatening arrhythmias (Snow 1965; Norris et al. 1968). Long-term outcome of morbidity and mortality suggested a beneficial effect even when not initiated immediately following the event. In fact, one of the first randomised study with propranolol had to be terminated early because of significantly better outcome in the verum than the placebo group (Beta-Blocker Heart Attack Trial 1982, 1983). Hence in the absence of any contraindications to β-blockade, long-term use of propranolol was recommended after a myocardial infarction, and as outlined above, severe heart failure had been a clear contraindication. Meta-analysis of numerous similar clinical studies confirmed the beneficial long-term effects of β-blockers on mortality and morbidity (Yusuf et al. 1985). Several small trials also investigated whether short-term treatment was of benefit but with contradicting results. A reliable answer was eventually given by the International Study of Infarct Survival group who investigated outcome in patients with myocardial infarction both after initiation of treatment during the acute phase and during long-term therapy for secondary prevention. Atenolol applied on top of digoxin and diuretics within 5 h after the suspected acute event, first intravenously followed by oral administration, significantly reduced mortality and rehospitalisation (ISIS-1 1986). Again, severe heart failure was an exclusion criterion.

The paradigm that β-blockers could only be administered safely in the absence of heart failure persisted for quite some time after Waagstein’s daring therapy, despite fundamental changes in management of suspected or acute myocardial infarction and secondary prevention. These changes comprised revascularization, implantable, and wearable cardioverting devices and new groups of drugs such as antiplatelet drugs, modifiers of the renin–angiotensin–aldosterone system (RAAS), lipid-lowering statins, mineralocorticoid receptor antagonists, β-blockers, and inhibitors of sodium-glucose cotransporter-2 (SGLT2). Beta-blockers reduced morbidity and mortality when administered on top of angiotensin converting enzyme (ACE) inhibitors or angiotensin receptor antagonists, which on their own were effective for treatment or prevention of cardiac decompensation post-myocardial infarction by ameliorating excessive neurohumoral activation.

Interestingly, the 2012 ESC guidelines for management of acute myocardial infarction advocated *oral* treatment with β-blockers in patients with heart failure or LV dysfunction (class 1 recommendation) but advised *intravenous* administration *only* in patients with no signs of heart failure who presented with high blood pressure and/or with tachycardia and without any contraindications to β-blockers (Steg et al. 2012). In contrast, the 2023 ESC guidelines recommend β-blockers in long-term management of patients with acute coronary syndrome/myocardial infarction, when LVEF is 40% or lower, regardless of heart failure symptoms (Byrne et al. 2023). Thus, Waagstein’s successful treatment with β-blockers following myocardial infarction has been confirmed in large clinical studies. Paradoxically, patients with mildly reduced or even preserved ejection fraction appear to benefit less from β-blocker therapy after myocardial infarction (Joo 2023). Taken together, today consensus exists that long-term β-blocker therapy following myocardial infarction in patients with *impaired* EF reduces mortality and hospitalisations for worsening of heart failure, whereas a similarly favourable effect is absent in patients with *preserved* EF (Joo 2023; Gomes et al. 2025). Therefore, heart failure with ventricular dysfunction can no longer be regarded as an absolutely contraindication for β-blockers, at least not in the context of myocardial infarction. For a critical discussion of the open questions concerning β-blocker therapy in secondary prevention post-MI, the reader is referred to an excellent recent review of the topic (Cataldo Miranda et al. 2025).

## Follow-up of evidence-based treatment of congestive heart failure

Despite the evidence provided by Waagstein and coworkers for the beneficial effects of β-blockers on heart failure symptoms in decompensated ischemic cardiomyopathy (Waagstein et al. 1975), it took two decades to establish these drugs as standard treatment of congestive heart failure (CHF). One reason may have been the absence of large clinical trials due to lack of financial resources. Instead, clinical trials concentrated on adding vasodilators on top of diuretics and digoxin, since patients with CHF often exhibited increased peripheral vascular resistance. The positive outcome of a trial with hydralazine and isosorbide nitrate (Cohn et al. 1986) led to two large studies with the ACE inhibitor enalapril as vasodilator in severe heart failure (CONSENSUS 1987; SOLVD 1990). Interestingly, enalapril on top of standard therapy significantly reduced mortality but only when it was due to worsening of heart failure and not when due to arrhythmia (SOLVD 1990).

Based on the available evidence in the beginning of the 1990 s, Teo and coworkers (1992) evaluated the at-that-time common management of CHF. ACE inhibition was confirmed to be effective in reducing mortality and morbidity in severe left ventricular dysfunction and CHF, and other systemic vasodilators were considered likely to be also beneficial. The effects of digitalis on survival and morbidity in CHF were under scrutiny, whereas other inotropic agents, at least in the long term, were shown to be clinically detrimental. Diuretics decreased morbidity, but their effect on mortality in CHF was still unknown. Although offering some promise, β-blocker therapy still had to await definitive clinical trials for evaluation (Teo et al. 1992).

Current guideline-recommended medical therapy of heart failure with reduced ejection fraction (HFrEF) with EF of ≤ 35% includes the following groups of drugs: modulators of RAAS (ACE inhibitors, ATRA, and angiotensin receptor–neprilysin inhibitors [ARNIs]), β-blockers, mineralocorticoid receptor antagonists (MRAs), and sodium-glucose co-transporter-2 (SGLT2) inhibitors (McDonagh et al. 2021, 2023; Heidenreich et al. 2022). Retrospective evaluation of the novel antidiabetic glucagon-like peptide-1 receptor agonist liraglutide showed promising survival benefits in HFrEF and HF with preserved EF (HFpEF) (Chen et al. 2025), but this finding warrants further investigation. Incidentally, evidence is accumulating that favourable effects of β-blocker therapy are questionable in patients with HFpEF (Wernhart et al. 2023), which is similar to the lack of benefit from β-blocker treatment after myocardial infarction in this group of patients (Joo 2023; Gomes et al. 2025).

The current American and European guidelines for treatment of acute and chronic heart failure recommend four β-blockers: metoprolol succinate (controlled release/extended release, CR/XL), carvedilol, bisoprolol, and nebivolol (Heidenreich et al. 2022; McDonagh et al. 2023). The relevant clinical trials that provided the necessary evidence are listed in Table 1. Interestingly, the COPERNICUS study of carvedilol in *severe* heart failure (Packer et al. 2002) was published more than a quarter century after Waagstein’s initial report of successful treatment with alprenolol (Waagstein et al. 1975). The table also includes the study with bucindolol, despite the fact that no survival benefit was observed with this β-blocker (Eichhorn et al. 2001; Metra et al. 2001). By adjusting for differences in the patient cohorts of these seminal trials, Domanski and co-workers arrived at the conclusion that different heart failure population subgroups may possibly have different responses to β-blocker therapy (Domanski et al. 2003).

**Table 1:** Clinical trials for use of β-blockers in congestive heart failure

β-Blocker	Acronym	Title	Reference
Metoprolol	MERIT-HF	Effect of metoprolol CR/XL in chronic heart failure: Metoprolol CR/XL Randomised Intervention Trial in Congestive Heart Failure	(Merit-Hf 1999)
		Effects of controlled-release metoprolol on total mortality, hospitalizations, and well-being in patients with heart failure: the Metoprolol CR/XL Randomised Intervention Trial in congestive heart failure (MERIT-HF). MERIT-HF Study Group	(Hjalmarson et al. 2000)
Bisoprolol	CIBIS-II	The Cardiac Insufficiency Bisoprolol Study II (CIBIS-II): a randomised trial	(Cibis-II 1999)
Carvedilol	U.S. Carvedilol Heart Failure Study Group COPERNICUS	The effect of carvedilol on morbidity and mortality in patients with chronic heart failure	(Packer et al. 1996)
		Effect of carvedilol on the morbidity of patients with **severe** chronic heart failure: results of the carvedilol prospective randomised cumulative survival (COPERNICUS) study	(Packer et al. 2002)
Nebivolol	SENIORS	Randomised trial to determine the effect of nebivolol on mortality and cardiovascular hospital admission in elderly patients with heart failure (SENIORS)	(Flather et al. 2005)
Bucindolol*^)^	BEST *^)^ No significant survival benefit	A trial of the beta-blocker bucindolol in patients with advanced chronic heart failure	(Eichhorn et al. 2001)

Since all drug classes of guideline-recommended medical therapy exhibit evidence-based benefit, the question arises in which order they should be started. Packer and coworkers (2024) concluded that the specific order does not matter, as long as treatment with these foundational drugs is initiated within weeks of first diagnosis. Low starting doses seem to improve outcome, but especially for β-blockers, up-titration to target doses in rapid sequence increases mortality benefits (Packer and McMurray 2021; Packer et al. 2024). It should be noted that the beneficial effect of guideline-recommended medical therapy for de novo HFrEF may require several weeks or even months to develop, so that patience is required while waiting for reverse remodelling (Borovac 2024).

## Outlook

Results from clinical studies vary by nature of inherent differences in study design, patient cohorts, and also differences in pharmacological properties of individual β-blockers. In addition to exhibiting diverse ancillary actions, β-blockers show distinct patterns of interactions with their receptors including subtype-selectivity and antagonistic *versus* (inverse/biased) agonistic properties (for excellent review, see (Brand et al. 2024)). The signal-transduction cascades involved in β-adrenergic stimulation are known to adapt during disease development (Bristow 2000) and novel, but not undisputed signalling patterns have been described (Benkel et al. 2022; Lefkowitz et al. 2023). These research efforts also aim at defining novel drug targets for treating heart failure fine-tune GPCR signalling as illustrated by inhibitors of G protein-coupled receptor kinases (Pfleger et al. 2019; Schmid et al. 2015). This enzyme family is responsible for desensitizing β-adrenergic receptors during prolonged stimulation, as for instance in the failing heart, and inhibiting their activity, esp. GRK-2 and GRK-5, with the small molecules was shown to improve contractile function in models of heart failure. The paroxetine derivative CCG258208 seems to represent such a promising new GRK-2 inhibitor (Gao et al. 2025; Roy et al. 2025). In addition, newly discovered extra-cardiac effects on, e.g. inflammation, metabolism, and oxidative stress could also modify clinical outcome.

For almost two decades, Waagstein’s bold questioning of a therapeutic paradigm, despite being based on thorough pathophysiological observations in animals, was heavily opposed by the scientific community. His perseverance and untiring scientific research finally enforced acceptance and was even superseded by novel drugs tailored to specific steps in the signalling cascade of β-adrenergic receptors that may lead to a more personalised and effective approach to heart failure therapy, even beyond the use of β-blockers (Brand et al. 2024).

## Data Availability

All source data for this work (or generated in this study) are available upon reasonable request.
